# Exenatide improves cardiovascular risk factors in obese patients with type 2 diabetes mellitus: a prospective study

**DOI:** 10.3906/sag-2004-154

**Published:** 2021-02-26

**Authors:** Derya KÖSEOĞLU, Salih Suha KOPARAL, Özden ÖZDEMİR BAŞER, Dilek BERKER

**Affiliations:** 1 Department of Endocrinology and Metabolism, Erol Olçok Education and Research Hospital, Çorum Turkey; 2 Department of Radiology, Ankara City Hospital, Sağlık Bilimleri University, Ankara Turkey; 3 Department of Endocrinology and Metabolism, Yozgat State Hospital, Yozgat Turkey; 4 Department of Endocrinology and Metabolism, Ankara City Hospital, Sağlık Bilimleri University, Ankara Turkey

**Keywords:** Carotid intima media thickness, hsCRP, nonalcoholic fatty liver disease, subcutaneous fat thickness, visceral fat volume, lipid profile

## Abstract

**Background/aim:**

The aim of this study was to evaluate the effects of a 6-month treatment regimen with exenatide on the lipid profile, high-sensitivity C-reactive protein (hsCRP), carotid intima media thickness (CIMT), visceral adiposity, and nonalcoholic fatty liver disease (NAFLD), all of which are important cardiovascular risk factors.

**Materials and methods:**

This study included 45 obese patients with type 2 diabetes mellitus (T2DM). Baseline clinical findings, laboratory parameters, and ultrasonography findings were recorded. An exenatide recipe was given twice daily to the patients and, after 6 months of therapy, the same variables were compared. The compared parameters were lipid profiles, hsCRP, aspartat aminotransferase, alanine aminotransferase, gamma-glutamyl transferase, liver craniocaudal diameter, visceral fat volume, subcutaneous fat thickness, and CIMT. Liver diameter, visceral fat volume, subcutaneous fat thickness, and CIMT were measured by ultrasonography.

**Results:**

After therapy, statistically significant improvements were achieved in lipid profile, hsCRP, liver enzymes, body mass index, and waist and hip circumferences. Also, statistically significant decreases were obtained in liver craniocaudal diameter, subcutaneous fat thickness, visceral fat volume, and CIMT. The reduction of CIMT and liver diameter were not correlated with BMI and HbA_1c_ reduction.

**Conclusion:**

This study showed improvement in lipid profile and hsCRP levels with exenatide treatment. We also showed decrease in both visceral fat volume and subcutaneous fat thickness. We demonstrated significant decrease in liver enzymes with significant decrease in liver diameter. These findings support the use of exenatide in patients with NAFLD and T2DM. Additionally, this study showed that exenatide treatment given twice daily reduces CIMT in obese T2DM patients.

## 1. Introduction

Type 2 diabetes mellitus (T2DM) is an important risk factor for cardiovascular morbidity and mortality [1]. Patients with T2DM also frequently exhibit other cardiovascular risk factors like high blood pressure, dyslipidemia, nonalcoholic fatty liver disease (NAFLD), obesity, and visceral adiposity [2–5]. Because of the importance of cardiovascular events in T2DM, clinicians should focus on these cardiovascular risk factors. Considering these facts, the treatment of T2DM should include the aforementioned cardiovascular risk factors. 

Exenatide, a glucagon-like peptide-1 (GLP-1) analogue, is a commonly used drug for the treatment of T2DM [6]. Exenatide also has beneficial effects other than glisemic control. Obesity is an important factor in patients with T2DM, and exenatide has a well-known effect on weight reduction [7]. Limited studies have shown the favorable effects of exenatide on NAFLD, visceral adiposity, and inflammatory markers, all of which are important cardiovascular risk factors [8–11]. A prospective study showed that exenatide had a better hepatic protective effect than intensive insulin therapy in patients with NAFLD with elevated liver enzymes and T2DM [10]. Du et al. showed, with computed tomography measurement, that exenatide decreased visceral adipose tissue without a reduction in subcutaneous adipose tissue volume [9]. Carotid intima-media thickness (CIMT) is a marker of atherosclerosis and predicts cardiovascular events [12]. The effect of exenatide on CIMT has not been widely investigated to date. An animal study showed that local subcutaneous injections of exenatide reduced CIMT in diabetic rats [13] and, recently, it was shown that exenatide given once weekly significantly improved CIMT [14].

Exenatide reduces tryglyceride levels and increases high-density lipoprotein (HDL), but its effect on low-density lipoprotein (LDL) was found to be controversial in some studies [9,11]. High-sensitivity C-reactive proteins (hsCRP) are another marker of cardiovascular risk prediction, and they decreased after exenatide therapy in several studies [15].

In this prospective study, we aimed to evaluate the effects of a 6-month treatment regimen with exenatide on lipid profile, hsCRP levels, CIMT, visceral adiposity, and some NAFLD parameters. 

## 2. Methods

This study was a prospective study and included patients with T2DM who were admitted to our center between 2015 and 2017. All of the patients included in this trial had a body mass index (BMI) over 35 kg/m^2^, were over 18 years old, and were not using exenatide before the study. All patients were using metformin with/without sulfanylurea at the beginning of the study. The indication for exenatide treatment was to achieve glycemic control and to reduce weigh for better cardiovascular outcomes. Patients who had drug or dose changes in the 6 months since the study began were excluded. All patients continued their prior medical therapy for diabetes, hypertension, and hyperlipidemia throughout the study period, and patients who needed a new drug or stopped a medication for any reason were excluded from statistical analysis. Patients who had recent cerebrocardiovascular events, renal failure, severe chronic infections, neoplasms, cardiovascular diseases (hypotension, uncontrolled hypertension, or Takayasu arteritis), a history of acute pancreatitis, alcohol consumption, cirrhosis, and chronic hepatitis were excluded from the study. Patients who were pregnant or intended to become pregnant were not included in the study. All patients underwent laboratory analysis before the treatment, and patients with the following parameters were excluded from the study: higher LDL levels than 200 mg/dL, higher triglyceride levels than 400 mg/dL, lower HDL levels than 30 mg/dL, and higher aspartate aminotransferase (AST) or alanin aminotransferase (ALT) levels than 100 U/L. Patients who needed a dose change of antihypertensive or antihyperlipidemic drugs during the study were also excluded from the statistical analysis. All patients with an indication for exenatide and who had no exclusion criteria were included in the study. After inclusion in the study, exenatide, at a dose of 5 μg twice a day, was given, and the dose was increased to 10 μg twice daily after 4 weeks. A standard hypocaloric diabetic diet and regular standard aerobic exercise were prescribed to all patients. The patients continued their daily dietary and exercise routines without any limitation.

In terms of baseline demographic data, clinical characteristic features, and duration of T2DM, comorbidities and concomitant medications were recorded. Weight and height were measured, and BMI was calculated as the weight (kg) divided by the square of the height (m) at the beginning and after the 6 months of treatment. All patients underwent laboratory analysis before treatment and after the 6 months of the study participation. Fasting blood samples were collected between 08.00 and 09.00. The measures recorded in the laboratory analysis included complete blood count (CBC), creatinine, ALT, AST, gamma-glutamyl transferase (GGT), fasting plasma glucose (FPG), postprandial plasma glucose (PPG), glycated hemoglobin (HbA_1c_), albumin, LDL, HDL, triglyceride, and hsCRP. 

Ultrasonography (US) (Toshiba Aplio Ultrasound Imaging System, Japan) was performed at the beginning of the study and after 6 months of exenatide treatment. After an overnight fasting period, visceral fat was measured in the supine position. For each patient, 4 measurements were obtained: 1) the distance between the internal surface of the splenic vein and the abdominal muscle; 2) the distance between the internal surface of the abdominal muscle and the posterior wall of the aorta on the umbilicus; 3) the thickness of the fat tissue of the posterior right renal wall in the right posterior perinephric space; and 4) the thickness of preperitoneal and subcutaneous fat layers in the xiphoid process [16]. The thickness of the preperitoneal and subcutaneous fat layers in the xiphoid process was determined as subcutaneous fat thickness. The visceral fat volume was calculated as: [visceral fat volume] = –9.008 + 1.191 × [distance between the internal surface of the abdominal muscle and the splenic vein (mm)] + 0.987 × [distance between the internal surface of the abdominal muscle and the posterior wall of the aorta on the umbilicus (mm)] + 3.644 × [thickness of the fat layer of the posterior right renal wall (mm)] [16]. A 7.5-MHz linear-array probe was used to measure the thickness of the subcutaneous and the preperitoneal fat layers, and a 3.75-MHz convex-array probe was used to for all other measurements. The procedures were performed by the same investigator. The intraexamination coefficient of variation for US was 1%. To assess the reliability and reproducibility of US, 5 participants were selected, and these measurements were repeated at the same time after 1 day by the same investigator. Within 95% confidence limits, the intraclass correlation coefficient was 97.6. This assessment showed the reproducibility and reliability of our measurements. CIMT was measured by US in the supine position by the same experienced radiologist. High-resolution B-mode US images (Logic 3, General Electric, USA), using an 11-MHz linear array transducer, were applied for CIMT measurements. Three arterial wall segments in each carotid artery were obtained from a fixed lateral transducer angle at the far wall. The mean CIMT was calculated from the 3 segments of both carotid arteries. During the same session, the liver was evaluated for lipid accumulation, and the maximum craniocaudal diameter of the liver was then measured. NAFLD was determined as having a diffuse hyperechoic texture of the liver in ultrasonography and when other reasons for steatosis, including alcohol consumption, drug use, and chronic hepatitis, are excluded. Liver biopsy was not performed in this study.

The Local Ethics Committee approved the study design, and methods and written informed consent were obtained from all participants included in this study.

Statistical analyses were performed with SPSS software 21.0 (SPSS, IBM, Armonk, NY, USA). The Kolmogorov–Smirnov test was performed to assess the normality of the distribution of data. Data with normal distribution variables were expressed as mean ± standard deviation (SD), and a paired sample t-test was used to compare values before and after therapy. Data without normal distribution were expressed as median (interquartile range), and a Wilcoxon test was used to compare values before and after therapy. Pearson’s correlation analysis was used to determine correlation. P-values less than 0.05 were considered statistically significant. 

## 3. Results

This study included 60 patients during the study participation, but the final statistical analysis was performed with 45 patients, including 41 women and 4 men. Eight patients dropped out of follow-up, and 7 patients had adverse events (nausea in 5 patients, drug eruption in 2 patients). The mean age of the study participants was 47.91 ± 7.30. All of the patients had been using antidiabetic drugs before the study, and their BMI were over 35 kg/m^2^. All of the patients were using metformin before the study participation, whereas 13 patients were using gliclazide with metformin.

The laboratory parameters before and after therapy are summarized in Table 1. Statistically significant decreases were achieved in triglyceride, total cholesterol, LDL, hsCRP, AST, ALT, and GGT levels. BMI, waist, and hip circumference values decreased significantly with exenatide therapy (Table 2). After exenatide therapy, statistically significant decreases were obtained in subcutaneous fat thickness (Figure a), visceral fat volume (Figure b), liver craniocaudal diameter (Figure c), and CIMT (Figure d) (Table 2). The mean decrease of visceral fat volume and subcutaneous fat thickness were 16.08% and 19.08%, respectively. The visceral fat volume/subcutaneous fat thickness ratio improved from 1.53 ± 0.24 to 1.92 ± 0.30 (P = 0.021). All of the patients had steatosis on US at the beginning of the study. A significant reduction in the craniocaudal liver diameter was achieved with treatment (Table 2).

**Figure:**
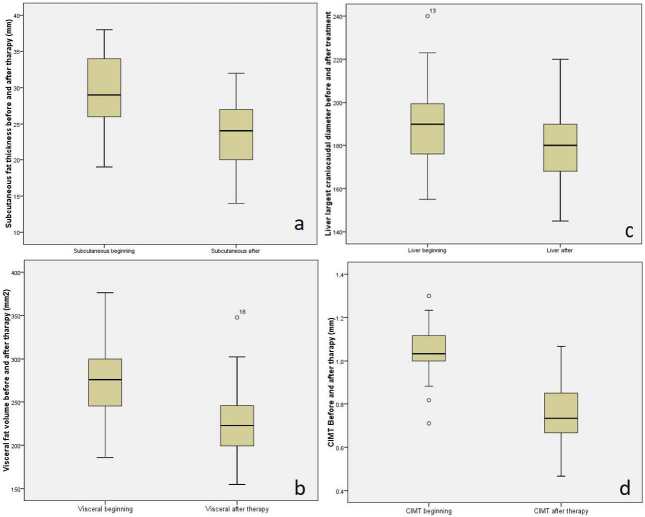
Values of subcutaneous fat thickness (a); visceral fat volume (b); liver largest craniocaudal diameter (c), and carotid intima media thickness (d), before and after exenatide treatment.

**Table 1:** Laboratory findings before and after exenatide therapy.

	Before exenatide treatment	After exenatide treatment	P-value
Triglyceride (mg/dL)	174.02 ± 85.52	145.47 ± 50.52	0.019
Total cholesterol (mg/dL)	211.8 ± 43.38	183.23 ± 32.40	<0.001
HDL (mg/dL)	48.77 ± 9.83	51.70 ± 9.62	0.034
LDL (mg/dL)	128.72 ± 38.64	103.00 ± 27.44	<0.001
non-HDL cholesterol (mg/dL)	162.81 ± 40.42	132.63 ± 32.34	<0.001
FPG (mg/dL)	155.13 ± 46.51	117.69 ± 26.10	<0.001
HbA_1c_ (%)	7.80 ± 1.31	6.71 ± 0.92	<0.001
hsCRP (mg/L)	8.66 ± 6.02	5.16 ± 2.85	<0.001
Creatinin (mg/dL)	0.77 ± 0.09	0.74 ± 0.10	0.074
ALT (U/L)	23 (10-99)	19 (11-51)	<0.001
AST (U/L)	20 (12-70)	19 (12-55)	0.005
GGT (U/L)	49.40 ± 17.75	33.78 ± 8.26	<0.001

HDL: high-density lipoprotein; LDL: low-density lipoprotein; FPG: fasting plasma glucose; HbA_1c_: glycated hemoglobin; ALT: alanin aminotransferase; AST: aspartate aminotransferase; GGT: gamma-glutamyl transferase.

**Table 2:** Anthropometric measures and ultrasonographic findings before and after exenatide therapy.

	Before exenatide treatment	After exenatide treatment	P-value
BMI (kg/m^2^)	43.46 ± 6.01	39.60 ± 5.63	<0.001
Waist circumference (mm)	126.47 ± 15.10	120.49 ± 15.24	<0.001
Hip circumference (mm)	132.82 ± 12.86	125.96 ± 12.39	<0.001
Liver largest craniocaudal diameter (mm)	188.26 ± 17.41	180.09 ± 16.49	<0.001
Subcutaneous fat thickness (mm)	29.10 ± 5.11	23.49 ± 4.98	<0.001
Visceral fat volume (mm3)	271.52 ± 41.01	227.54 ± 39.42	<0.001
CIMT (mm)	1.04 ± 0.11	0.75 ± 0.12	<0.001

BMI: body mass index; CIMT: carotid intima-media thickness.

Correlation analysis was performed, and the reduction of HbA_1c_, weight, CIMT, liver diameter, subcutaneous fat thickness, and visceral fat volume were included in the analysis (Table 3). The reduction of CIMT did not correlate with BMI and HbA_1c_ reduction. The reduction of subcutaneous fat thickness and visceral fat volume both correlated with weight reduction but not with HbA_1c_. The reduction of liver diameter only correlated with subcutaneous fat thickness reduction but not with HbA_1c_ and weight reduction.

**Table 3:** Correlation analysis of the changes of HbA_1c_, weight, CIMT, subcutaneous fat thickness, and visceral fat volume.

	ΔCIMT	ΔSubcutaneous fat thickness	ΔVisceral fat volume	ΔHbA_1c_	Δweight	Δliver diameter
ΔCIMT		r : 0.262P : 0.099	r : 0.384 P : 0.013	r : 0.214 P : 0.173	r : 0.130 P : 0.405	r : -0.055 P : 0.724
ΔSubcutaneous fat thickness	r : 0.262P : 0.099		r : 0.486 P : 0.001	r : 0.227 P : 0.170	r : 0.395 P : 0.013	r : 0.434 P : 0.002
ΔVisceral fat volume	r : 0.384 P : 0.013	r : 0.486P : 0.001		r : 0.155 P : 0.353	r : 0.373 P : 0.019	r : 0.252P : 0.087
ΔHbA_1c_	r : 0.214 P : 0.173	r : 0.227 P : 0.170	r : 0.155P : 0.353		r : 0.486 P : 0.001	r : 0.222 P : 0.164
ΔWeight	r : 0.130P : 0.405	r : 0.395P : 0.013	r : 0.373 P : 0.019	r : 0.486P : 0.001		r : 0.225 P : 0.146
ΔLiver diameter	r : -0.055 P : 0.724	r : 0.434P : 0.002	r : 0.252P : 0.087	r : 0.222 P : 0.164	r : 0.225 P : 0.146	

Weight reduction was achieved in 44 patients, and 1 patient had no change in weight. Thirty-five patients had a weight reduction of more than 5%. When patients with less than 5% weight reduction were included in the analysis, CIMT, liver diameter, visceral fat volume, and subcutaneous fat thickness still showed statistically significant decreases (P = 0.005, P = 0.005, P = 0.008, P = 0.008, respectively). 

## 4. Discussion

In this study, we investigated the effects of 6 months of exenatide treatment on some cardiovascular risk factors and showed that exenatide had favorable effects on lipid profile, hsCRP, NAFLD, CIMT, visceral adiposity, and subcutaneous adiposity.

We observed significant improvement in glucose control, with a significant reduction of HbA_1c_ and fasting blood glucose from baseline. This was consistent with the existing literature [17]. Additionally, exenatide treatment was associated with a significant reduction of triglyceride, LDL, non-HDL cholesterol, and a significant increase in HDL cholesterol levels. Studies showed increases in HDL levels, but the effect of exenatide on LDL varies in different studies [9,11,18]. Our study showed statistically significant decreases in LDL and non-HDL cholesterol. The discrepancy may be due to patient selection. Our study included only obese patients whose BMI was over 35 kg/m^2^. 

Weight reduction is a well-known effect of exenatide [7,17,19]. Abdominal adipose tissue consists of visceral and subcutaneous adipose tissues, which have different functions [9]. Visceral adipose tissue is an important cardiovascular risk factor and also considered bad fat [9,20]. Computed tomography (CT), magnetic resonance imaging, and US are the methods used for measuring adiposity. Hirooka et al. showed that measuring visceral fat volume with US had similar results with CT and may be used effectively [16]. We used US in our study because of the advantage of its low cost and because it had no side effects. Our study showed statistically significant decreases in both visceral fat volume and subcutaneous fat thickness. Even when only patients with weight reduction less than 5% were included in the analysis, significant decreases in visceral fat volume and subcutaneous fat thickness persisted. Limited evidence is available about the effect of exenatide on visceral adiposity. Using CT measurements, Du et al. [9] showed that exenatide decreased visceral adipose tissue without a statistically significant decrease in subcutaneous fat volume. They also mentioned that the visceral/subcutaneous fat volume decreased with exenatide [9]. This was not in accordance with our findings because we found statistically significant decreases in both visceral and subcutaneous fat tissues and, furthermore, the decrease of subcutaneous fat thickness was more apparent. The visceral fat volume/subcutaneous fat thickness ratio increased significantly. To the best of our knowledge, our study showed for the first time that the visceral/subcutaneous fat ratio increases with exenatide therapy. Our study included only obese patients over BMI of 35 kg/m^2^, and our findings should be supported with further studies with broad selection criteria. In our study group, hsCRP, which is also a cardiovascular risk factor, decreased after 6 months of exenatide therapy. The reduction of hsCRP with exenatide was previously reported on, and our study supports this finding [15]

NAFLD, T2DM, and obesity are commonly seen simultaneously in patients, and the prevalence of NAFLD reaches 75% in patients with T2DM [21]. Patients with NAFLD are at an increased risk for cardiovascular events [22,23]. Because NAFLD is an important cardiovascular risk factor, it is important to treat it. However, the treatment of NAFLD remains inadequate, and it is generally directed towards weight loss and comorbidity treatment [24]. GLP-1 receptor agonists have potential beneficial effects on NAFLD. Liraglutide, a GLP-1 receptor agonist, has beneficial effects on NAFLD, as well [25,26]. Exenatide has not been widely investigated in patients with NAFLD and T2DM, and few studies have shown the promising effects of exenatide on NAFLD [10,27]. We showed in our trial that AST, ALT, and GGT levels decreased with exenatide treatment; we also showed that the liver craniocaudal diameter significantly decreased with exenatide treatment. Furthermore, the decrease of liver diameter was not correlated with weight reduction and HbA_1c_ reduction. US is a first step in the evaluation of NAFLD but has no effectiveness in differentiating NAFLD from nonalcoholic steatohepatitis (NASH). The liver biopsy is still the gold standard for evaluating NAFLD and NASH, but it has disadvantages, including cost, sampling error, inter–observer variability, and procedure-related complications [28]. The sensitivity of US on NAFLD is low, but the specificity of US in predicting NAFLD was found to be 94% in a metaanalysis [29]. A limitation of our study is the lack of a liver biopsy, but obtaining a liver biopsy is beyond the aim of this study. This limitation prevented in giving strong advice about the use of exenatide on NAFLD, but our findings may be a guide for further studies. A further study with a liver biopsy before and after exenatide therapy may show clinical benefits more clearly.

CIMT is a marker of atherosclerosis and predicts cardiovascular events [12,30]. CIMT is a noninvasive and effective method predicting atherosclerosis, and it is easily measured with ultrasonography. Recently, it was shown that administering exenatide once weekly improved CIMT for the first time in the literature [14]. A more recently published study showed that exenatide treatment could prevent atherosclerosis progression in patients with T2DM, compared to insulin therapy [31]. We showed that exenatide treatment for 6 months significantly decreased CIMT in obese patients with T2DM. Moreover, the reduction of CIMT did not correlate with HbA_1c_ reduction and weight reduction. The decrease of visceral fat volume correlated with CIMT reduction, which also supports the fact that visceral fat predicts cardiovascular risk [32]. The potential effect of exenatide on such cardiovascular risk parameters may be varied. Obesity is in accordance with CIMT, and weight reduction may be a reason for the CIMT reduction in our study [33]. However, our study showed no correlation between weight reduction and CIMT reduction, and even patients with less weight reduction had a significant decrease in CIMT. These findings suggest that mechanisms other than weight reduction also have effects on the decrease of CIMT. It has been shown that exenatide reduces oxidative stress and has beneficial effects on antioxidant enzymes in human in vitro monocytes/macrophages [34]. Another study showed that exenatide improved diastolic function and reduced arterial wall stiffness [35]. Further studies are needed to show the exact mechanism of exenatide on CIMT and atherosclerosis. 

Our study included only obese patients with a BMI over 35 kg/m^2^ because of the national health care system reimbursement conditions. This is a limitation of our study, and our findings cannot be generalized to the whole population. Studies including patients with a BMI less than 35 kg/m^2^ should be performed to assess our finding in the general population with T2DM. However, this limitation is also a positive aspect of the study because it may provide for the strong statistical findings in our study group. Another limitation is the study duration; a longer study duration could have resulted in more beneficial results, but the limitations do not hinder our positive results. The strengths of this study include the real world setting, blinded measurements of CIMT, liver diameter, and visceral and subcutaneous fat measurements. 

## 5. Conclusion

Our study showed that 6 months of exenatide treatment has beneficial effects on lipid profile, visceral fat volume, NAFLD, CIMT, and hsCRP, which are all important cardiovascular risk factors. Our study showed for the first time in the literature that exenatide reduces both visceral fat volume and subcutaneous fat thickness. We showed that exenatide has favorable effects on NAFLD beyond weight reduction. 

**Conflict of interest**

The authors declare no conflict of interest.

**Informed consent**

The Local Ethics Committee approved the study design and methods, and written informed consent was obtained from all participants included in this study. The approval date and number are 15 June 2016 and 16-963, respectively.
